# Near fatal anaphylaxis at general anesthesia in an 11 year old boy

**DOI:** 10.1186/2045-7022-3-S3-P33

**Published:** 2013-07-25

**Authors:** M Wickman, L Heise Garvey, M Mörrby Ramberg

**Affiliations:** 1 Institute of Environmental Medicine, Karolinska Institutet, Stockholm, Sweden; 2 Danish Anaesthesia Allergy Centre, Copenhagen University Hospital, Gentofte, Denmark; 3 Department of Anaesthetiology, South General Hospital, Stockholm, Sweden

## Background

Perioperative allergic reactions in children are rare. Investigations should ideally follow standardized protocol and include all drugs and substances used, since it is not possible to guess the causative agent. The aim of this case report is to increase awareness of agents used in relation to anesthesia and to show possible pathways for the identification of the causative agent.

## Methods

During general anaesthesia exposure to a number of different agents is common. In addition, anti-inflammatory drugs, antiemetics, antacids, antibiotics and analgesics may be used, and all may elicit reactions. Follow up is recommended to include serum tryptase, specific IgE, skin prick test (SPT), intradermal tests (IDT), basophil activation test (BAT) and provocations. We present a case of severe anaphylaxis in an 11-year-old boy during hand surgery with rhinitis at exposure to pollen and pets only.

## Results

Chlorhexidine was applied to his arm and anaesthesia was induced with propofol and fentanyl. A laryngeal mask was inserted and sevoflurane was used for maintenance of anaesthesia. A diclofenac suppository was administered and beclometasone, perfalgan (paracetamol) and ondansetron given intravenously. 35 min after induction tidal volume was reduced from 264 ml to 40 ml and s Systolic blood pressure dropped to 44-55 mmHg. Intubation was attempted, but failed and for 5 min O_2_ saturation was not measurable. Emergency treatment was promptly inducedHe developed facial edema was noted. After some improvement fiberoptic examination by an ENT specialist revealed profound edema of the tongue base and mild laryngospasm. Serum tryptase was 43 ug/L 2 hours after reaction. He recovered uneventfully.

Investigations revealed normal baseline tryptase of 3.2 ug/L. IgE to rocuronium, polyhexanide, suxamethonium, morphine, fholcodine, chlorhexidine, ethylene oxide and latex all negative. BAT for propofol, fentanyl, ondansetron and diclofenac all negative. He was multisensitized to pollens and pets. At examination exhaled NO was 53 ppb, spirometry normal.

## Conclusion

Clinical reaction and elevated serum tryptase indicate an immunologic reaction. BAT is planned for remaining drugs. SPT (and IDT if possible) is planned for diclofenac, propofol, fentanyl, ondansetron, chlorhexidine, beclomethasone and perfalgan. In addition provocations will be performed aiming to make future anaesthetics safe by identifying the causative agent and proving tolerance to the remaining agents.

## Disclosure of interest

None declared.

